# Fibroids and pregnancy

**DOI:** 10.1002/ijgo.70612

**Published:** 2025-11-06

**Authors:** Diana Ramasauskaite, Nikhil Purandare, Ivonne Diaz, Greta Kvederaite‐Budre, Titus K. Beyuo, Jolly Beyeza‐Kashesya, Bo Jacobsson

**Affiliations:** ^1^ Clinic of Obstetrics and Gynecology Vilnius University Faculty of Medicine Vilnius Lithuania; ^2^ Department of Obstetrics and Gynaecology University College Hospital Galway, University of Galway Galway Ireland; ^3^ Department of Obstetrics and Gynecology Nueva Granada and Unisanitas University Bogotá Colombia; ^4^ Department of Obstetrics and Gynaecology University of Ghana Medical School Accra Ghana; ^5^ Department of Obstetrics and Gynecology Mulago Specialised Women and Neonatal Hospital Kampala Uganda; ^6^ Department of Obstetrics and Gynecology Sahlgrenska Academy, University of Gothenburg Gothenburg Sweden; ^7^ Department of Obstetrics and Gynecology Sahlgrenska University Hospital Gothenburg Region Västra Götaland Sweden; ^8^ Department of Genetics and Bioinformatics, Division of Health Data and Digitalisation Institute of Public Health Oslo Norway

**Keywords:** complications, fibroids, leiomyoma, management, pregnancy

## Abstract

The prevalence of uterine fibroids in pregnancy varies between 1.6% and 10.7%. Pregnancies involving uterine fibroids are generally uncomplicated. However, complications can occur, particularly in cases of multiple fibroids, when the fibroids are larger than 5 cm, or when they are located in the lower uterine segment. Between 10% and 30% of pregnant women with fibroids experience complications during pregnancy, labor, and the postpartum period. The most common complication during pregnancy, which can occur in 8% of women, is red degeneration. High‐quality data on the relationship between fibroids and pregnancy outcome are very limited. Potential obstetric complications include preterm birth (increased risk, OR 1.5; 95% CI, 1.3–1.7), malpresentation of the baby (OR 2.65; 95% CI, 1.60–3.70), placental abruption (OR 2.63; 95% CI, 1.38–3.88), placenta previa (OR 2.21; 95% CI, 1.48–2.94), cesarean birth (OR 2.60; 95% CI, 2.02–3.18), postpartum hemorrhage (OR 2.95; 95% CI, 1.86–4.66), and other rare occurrences. Most women with uterine fibroids are able to deliver vaginally without complications. Cesarean birth is typically performed for standard obstetric indications. It is recommended that cesarean myomectomy should be avoided when possible.

## 1 INTRODUCTION

The prevalence of uterine fibroids in pregnancy varies between 1.6% and 10.7%^1^, ^2^ and is influenced by maternal age and race/ethnicity, among other factors. In one study, the prevalence of uterine fibroids in pregnancy was 12% in patients aged 19–25 years, increasing to 32% in patients aged 35–42 years.^2^ Furthermore, according to ethnicity, the highest prevalence of 14% was found in non‐Hispanic Black women compared with 9% in non‐Hispanic white women, 6% in Hispanic women, and 10% in Asian or Pacific Islander women.^2^ Maternal age is rising worldwide, which will lead to an increase in the prevalence of uterine fibroids.

FIGO recommends classifying the uterine submucosus fibroids as types 0 (pedunculated intracavitary), 1 (<50% located within endometrium), and 2 (≥50% located within the myometrium). Other fibroids are type 3 (contacts endometrium, but 100% located within the myometrium), type 4 (intramural), type 5 (subserosous, ≥50% within the myometrium), type 6 (subserosous, <50% within the myometrium), type 7 (subserosous, pedunculated), type 8 (cervical, parasitic). The fibroids from the hybrid category contact both the endometrium and the uterine serosa.^3^ Management of uterine fibroids prior to conception presents clinical challenges, particularly when they are asymptomatic, classified as type 3 or hybrid 2–5 (submucous or subserous fibroids, with more than half their mean diameter within the myometrium) and measure up to 5 cm. Pregnancy after a prior myomectomy poses challenges due to the increased risk of uterine rupture, placenta accreta spectrum disorders, and placenta previa, among others. Fibroids with possible red degeneration that do not respond to conservative treatment may necessitate emergency surgery during pregnancy. Complications associated with fibroids include preterm birth, malpresentation, placental abruption, placenta previa, cesarean birth, postpartum hemorrhage, and other rare occurrences. The aim of this article is to provide good clinical practice recommendations for managing pregnancy in patients with fibroids or a history of myomectomy.

A narrative review was conducted and a literature search was performed in databases such as PubMed, Scopus, and Web of Science for the last two decades. The MeSH terms used were “myoma/fibroid” AND “pregnancy clinical presentation”, “pregnancy complications”, “obstetrical complications”, “pregnancy ultrasound”, “pregnancy growth”, “evaluation during pregnancy”, “postpartum evaluation,” “delivery”. Additionally, a search was conducted using “myomectomy” AND “pregnancy”, “fertility”, “preconception”, “pregnancy outcome”, and “delivery”. We selected reviews, meta‐analyses, case series, clinical studies, and clinical guidelines published in English that provided full‐length articles. Findings from the narrative review are presented in the following sections.

## 2 PRECONCEPTION CONSULTATION AND MYOMECTOMY

Decisions on preconception myomectomy should be made individually, considering patient age, past reproductive history, severity of symptoms, and the size and location of fibroids. The age of women planning to become pregnant is increasing, leading to higher risk of infertility, fibroids, and early pregnancy loss. Fibroids may contribute to infertility and recurrent early pregnancy loss; however, existing data arise primarily from observational studies and often present conflicting results.^4^, ^5^ Long‐term medical therapies for symptomatic patients seeking fertility often lead to adverse effects and cause a rapid rebound of symptoms upon discontinuation, making them generally ineffective.^6^

For patients desiring fertility who present with heavy menstrual bleeding and a submucosal fibroid (FIGO type 0, type 1, or some type 2), hysteroscopic myomectomy is recommended for its minimally invasive relief of symptoms and optimization of fertility.^6^ The management of patients desiring to conceive who present with asymptomatic submucosal fibroids should be individualized. Patients with submucosal uterine fibroids who undergo hysteroscopic removal experience similar birth outcomes as those who do not have the procedure.^7^

For symptomatic patients desiring fertility who have fibroids other than submucosal, laparoscopic myomectomy (with or without robotic assistance) or an open abdominal incision is recommended. Selection of a laparoscopic or open abdominal technique depends on the number, size, and location of the fibroids, and the skill of the surgeon. Data are limited on pregnancy outcomes and the live birth rate is similar in both groups.^4^ Preconception management for patients with fibroids is summarized in Table 1.

**TABLE 1:** Preconception management of patients with uterine fibroids.

Type of fibroid	Asymptomatic patient	Symptomatic patient
Submucosal	Management should be personalized, as the pregnancy and delivery outcomes are comparable between individuals who undergo hysteroscopic removal and those who do not.	Hysteroscopic myomectomy
Other	Management should be individualized depending on patient age, fertility, and the number, size, and location of the fibroids	Laparoscopic myomectomy (with or without robotic assistance) or an open abdominal incision

Data from radiological findings show that uterine healing can be achieved after 3 months; however, other studies have shown that it may not be achieved until 6 months.^8^, ^9^, ^10^ Given that the likelihood of conceiving decreases with age, this raises the question of whether waiting longer is more advantageous to avoid uterine rupture during pregnancy and childbirth, or a disadvantage in terms of delaying conception. Delaying conception after surgery may increase uterine fibroid recurrence, which has been evaluated to be between 34.2% and 41.6% over 3 years.^11^

A systematic review showed that data are insufficient to advise a minimal time interval between myomectomy and conception to avoid uterine rupture.^12^ Patients who undergo myomectomy should be aware that uterine rupture could happen at any time during pregnancy, especially during the third trimester, and they should be informed about the clinical symptoms of this complication.^12^

When counseling patients about the appropriate waiting period after surgery before attempting conception, it is important to assess each case individually. Every surgical report should include the following specific information:

- Size and location of the fibroids
- Type of surgery performed
- Details about the operation (including whether energy was used, if the uterine cavity was entered, and the number of layers of stitches applied)
- Observations from the postoperative period

## 3 FIBROID CHANGES IN VOLUME AND SIZE DURING PREGNANCY AND POSTPARTUM

The growth of uterine fibroids during pregnancy is affected by increased pregnancy‐related steroid hormone levels and uterine blood flow. Increased levels of estrogen and progesterone during pregnancy are considered to play a crucial role in the growth of fibroids.^13^ The expression of estrogen receptors is higher in uterine fibroids than in adjacent myometrium.^14^ Sex steroids and other hormones secreted by the placenta and fetus, as well as their synergistic effects, may influence the growth of fibroids.^15^ Several studies have shown the effect of human chorionic gonadotropin (hCG) on fibroid growth, increasing the number of fibroid cells directly and through the effect of the prolactin secretion.^16^ This could explain why enlargement of fibroids occurs more frequently during early pregnancy. Higher pre‐pregnancy body mass index is associated with an increased risk of uterine fibroids enlarging during pregnancy, as adiposity affects hormonal and metabolic processes that influence fibroid growth.^17^

Smaller fibroids have greater potential to increase in size compared with larger fibroids during pregnancy.^2^, ^18^, ^19^ Fibroids with a diameter of 1–3 cm typically remain stable in size.^19^ Longitudinal studies have indicated that up to 71.4% of uterine fibroids increased in size between the first and second trimesters of pregnancy.^17^, ^19^ Fibroids typically stabilize in size during the second and third trimesters and reduce in size during the postpartum period.^18^, ^19^

The regression of fibroids is a common occurrence after labor. Fibroids decrease in volume by over 50% between early pregnancy and 3–6 months postpartum in 70% of women who have a live birth.^20^ The complex mechanical and cellular mechanisms associated with birth, including delivery of the placenta, hypoxia, vascular changes, hormonal changes, cellular apoptosis, and postpartum uterine involution may lead to regression.^21^ The greatest changes occur in submucosal fibroids (1.8 cm change), compared with intramural (0.2 cm), subserosal (0.6 cm), or pedunculated (0.5 cm) fibroids.^22^ Size changes depend on the location of the fibroids, as those in the lower segment are likely to show a greater change in diameter (1.4 cm) compared with those located in the corpus and fundus (0.5 cm and 0.4 cm, respectively).^22^

Lactation suppresses ovarian steroid production; therefore, due to the decreased exposure to hormones, fibroid size is stable or reduced during breastfeeding.^23^, ^24^

## 4 CLINICAL PRESENTATION AND PREGNANCY COMPLICATIONS

### 4.1 Clinical presentation of fibroids during pregnancy

Uterine fibroids are generally asymptomatic during pregnancy. However, in individuals who do experience symptoms, these may include pain, pelvic pressure, and/or vaginal bleeding. The most common symptom is pain, which tends to increase as the tumor grows larger. Other potential symptoms may include mild leukocytosis, fever, nausea, and vomiting. Symptoms typically emerge during the latter part of the first trimester and the early part of the second trimester, as the fibroid reaches its maximum size.^25^ The most common complication during pregnancy, which can occur in 8% of women, is red degeneration.^26^ This complication arises from acute peripheral venous thrombosis, leading to infarction and necrosis of the fibroid.^27^ Pregnant women with red degeneration often present with sudden focal abdominal pain, mild fever, nausea, vomiting, localized tenderness over the fibroid, and leukocytosis.^28^

Torsion is another complication of fibroids during pregnancy, with similar clinical features to red degeneration, and should be considered in patients who have a previously diagnosed pedunculated fibroid. Differentiation between these two conditions is crucial, as red degeneration is managed with conservative treatment, whereas torsion requires surgical intervention.^29^

Treatment for patients with painful fibroids typically begins with conservative management. Nonsteroidal anti‐inflammatory drugs (NSAIDs) are commonly recommended for initial treatment for up to 48 hours. In addition, hospitalization, intravenous fluids, and antiemetics may also be used.^25^ The US Food and Drug Administration (FDA) recommends avoiding using NSAIDs for pain relief after 30 weeks of gestation and limiting the dose to the lowest effective amount for the shortest duration between 20 and 30 weeks if treatment is deemed necessary^30^ to reduce the risk of early closure of ductus arteriosus, necrotizing enterocolitis, pulmonary hypertension and oligohydramnios. Low‐dose narcotic pain relief may be preferable to NSAIDs.^31^

While the management of uterine fibroids during pregnancy is primarily conservative, surgical intervention should be considered if torsion occurs or if medical treatment fails.

### 4.2 Obstetric complications

High‐quality data on the relationship between fibroids and pregnancy outcome are very limited.^25^ The research data indicate a slightly increased risk of complications, including early pregnancy loss, preterm birth, fetal malpresentation, and placental abruption. Most studies do not demonstrate an increased risk of adverse events.^32^

#### Early pregnancy loss

A meta‐analysis of nine cohort studies (>20 000 pregnant women) showed that there was no association between the presence of fibroids and pregnancy loss; however, the location of the fibroids was not considered in this study.^33^ The risk of pregnancy loss is likely higher in women with multiple fibroids compared with those with a single fibroid.^34^ Submucosal fibroids negatively impact implantation, placentation, and ongoing pregnancy. The effects of intramural fibroids are more controversial; however, fibroids that are primarily subserosal or pedunculated are unlikely to cause early pregnancy loss.^25^, ^35^

#### Preterm birth

Systematic literature reviews have reported an increased risk of preterm birth (unadjusted OR 1.5; 95% CI, 1.3–1.7;^35^ RR 1.43; 95% CI, 1.27–1.60)^36^ in pregnancies with uterine fibroids. Preterm birth is more frequent with multiple fibroids compared with single fibroids (18% vs. 6%; *P* = 0.05).^37^ Pregnant women with uterine fibroids larger than 5 cm have a significantly higher risk of delivering at an earlier gestational age compared with those with smaller or no fibroids.^38^ Although fibroids increase the risk of preterm birth, cervical length screening is not necessary if there are no additional risk factors.^25^

#### Placental abruption

Systematic literature reviews determined a significant association between uterine fibroids and placental abruption (OR 2.63; 95% CI, 1.38–3.88;^39^ OR 1.60; 95% CI, 1.20–2.14).^40^ Submucosal and retroplacental fibroids, and those with volumes greater than 200 mL (equivalent to diameters of 7–8 cm) are associated with the highest risk of placental abruption. These fibroids can cause abnormal blood flow to the placental site, which may lead to the separation of the placenta.^25^

#### Malpresentation

A meta‐analysis found a significant association between fibroids and malpresentation at term (OR 2.65; 95% CI, 1.60–3.70).^41^ Large submucosal fibroids that distort the uterine cavity, multiple fibroids, a fibroid located behind the placenta or in the lower uterine segment, and large fibroid (larger than 10 cm) are associated with a high risk of fetal malpresentation.^25^

#### Placenta previa

A meta‐analysis including 255 886 cases found a significant association between uterine fibroids and placenta previa (OR 2.21; 95% CI, 1.48–2.94); furthermore, the risk was higher with fibroids ≥5 cm.^42^

#### Cesarean birth

Results of meta‐analyses indicated that patients with uterine fibroids had a significantly higher risk of cesarean birth (OR 2.09; 95% CI, 1.69–2.58, *P* < 0.001;^40^ OR 2.60; 95% CI, 2.02–3.18).^41^ The location of the fibroid significantly influenced the birthing method, resulting in a higher cesarean birth rate for fibroids located in the lower segment of the uterus compared with those in the body of the uterus (86% vs. 40%; *P* = 0.01).^37^ An increase in cesarean births in patients with fibroids is mainly due to malpresentation, placenta previa, dysfunctional labor, obstruction of the birth canal, and fetal heart rate abnormalities related to abruption. However, a causal association remains unproven due to biases in the studies.^25^

#### Postpartum hemorrhage

Data from meta‐analyses indicate an increased risk of postpartum hemorrhage in pregnancies complicated by fibroids (unadjusted OR 1.8; 95% CI, 1.4–2.2;^35^ OR 2.95; 95% CI, 1.86–4.66).^40^ The location of the fibroid could have a significant effect on a higher rate of postpartum hemorrhage (22% vs. 11%; *P* = 0.03) and greater estimated blood loss (830 ± 551 mL vs. 573 ± 383 mL; *P* = 0.03) for fibroids in the lower part of uterus than in the body of the uterus.^37^ Blood loss at delivery was significantly higher for patients with large fibroids compared with patients with no fibroids or small fibroids (≤5 cm) (645.1 vs. 486.8 vs. 535.6 mL, respectively), as was blood transfusion (12.2% vs. 1.1% vs. 0.0%, respectively).^38^

#### Other complications

Obstetrical complications such as dysfunctional labor, fetal deformities (case reports on limb reduction defects, congenital torticollis, and head deformities), uterine incarceration, urinary tract obstruction with urinary retention or acute renal failure, puerperal uterine inversion, and pyomyoma—are extremely rare. In pregnancies complicated by fibroids, no increased risk is likely for complications such as preterm prelabor rupture of membranes, fetal growth restriction, fetal demise, and pre‐eclampsia.^25^

## 5 EVALUATION AND FOLLOW‐UP OF FIBROIDS DURING PREGNANCY

Ultrasound is the most suitable method for the initial evaluation of uterine fibroids. The first trimester is the best time for diagnosing and evaluating fibroids.^43^ After accurately determining their number, size, and location during the first trimester, monthly ultrasound checks should be conducted, as fibroid growth may alter the associated obstetric risk.^44^ It can be challenging to differentiate between fibroids and physiological thickening of the myometrium during ultrasound scans, especially when small fibroids are present. It is recommended to perform repeat scanning after 30 minutes and to use color Doppler at lower velocity settings.^44^, ^45^ Furthermore, Doppler is useful in assessing blood flow in fibroids, as the absence of blood flow may indicate a complication of the fibroids.^46^, ^47^ However, magnetic resonance imaging (MRI) has the greatest sensitivity for identifying the size, location, number, and extent of fibroid penetration into the myometrium.^48^, ^49^

MRI should be considered for differentiating between red degeneration and torsion of fibroids owing to its high diagnostic accuracy.^29^, ^50^ When a fibroid is suspected to be growing rapidly or when surgical management is considered, MRI can be useful by providing information about the anatomical position of the fibroid and its relationship to surrounding structures. MRI plays a vital role in characterizing fibroids, especially if a cesarean birth is required when fibroids are located in the lower anterior wall of the uterus. Usually, cesarean birth is reserved for standard obstetric indications. If a planned cesarean birth is considered, surgeons need to determine the incision location using ultrasonography and, if necessary, MRI.^51^

## 6 MYOMECTOMY DURING PREGNANCY

Surgery for asymptomatic fibroids is usually delayed after labor and breastfeeding. Conservative treatment is the preferred approach for managing symptomatic fibroids. Myomectomy is not routinely performed during pregnancy due to the risks of severe hemorrhage, which arise from increased uterine blood flow and volume during gestation, as well as potential complications such as uterine rupture, early pregnancy loss, and preterm birth.^52^ These risks may be heightened when removing submucous, posteriorly located, or multiple intramural myomas.^44^

Indications for myomectomy during pregnancy:^44^, ^52^

- Symptoms persist after 72 hours of conservative management
- Torsion of a pedunculated fibroid
- Severe compression of other pelvic organs by large myomas
- Rapid growth potentially hiding malignancy

A systematic review indicated that abdominal pain unresponsive to medical treatment was the most common reason for surgery, with necrosis and degeneration being the primary histopathological findings in the excised fibroids.^52^

Myomectomy can be performed during any trimester of pregnancy. The type of surgery should be individualized depending on the duration of pregnancy, location, type, and size of the fibroid, and other maternal factors. Laparotomic myomectomy has long been considered the first choice, and a systematic review found that laparotomy was performed in 78.4% of cases.^52^ Laparoscopic myomectomy could provide better intra‐abdominal visualization, a minimally invasive approach, and earlier mobilization after surgery. During the end of the second and third trimesters, abdominal access can be utilized with either the open technique or a Verres needle due to the size of the uterus. Carbon dioxide insufflation at 10–15 mmHg and bipolar electricity for hemostasis can be used safely.^44^

The left lateral position during surgery is recommended. Monitoring of fetal well‐being should be performed immediately before and after the surgery.

Transvaginal resection is reasonable in patients with a prolapsed fibroid and clinically significant bleeding, unmanageable pain, urinary retention, or infection.^53^ The procedure for transvaginal myomectomy depends on the type of fibroid (cervical or submucosal) and the thickness of the stalk/base, which can be determined clinically or by transvaginal ultrasound or MRI.^25^

## 7 LABOR CONSIDERATIONS RELATED TO FIBROIDS AND MYOMECTOMY

### 7.1 Mode of birth

Most women with fibroids are able to deliver vaginally without complications, with cesarean performed for standard obstetric indications. Planned cesarean birth may be considered for patients with large cervical fibroids or with lower uterine segment fibroids that deform the uterine cavity and are located between the fetal vertex and cervix in the third trimester.^37^, ^54^ If vaginal birth is chosen and there are no other indications to induce labor, the beginning of spontaneous labor could be expected.

### 7.2 Childbirth considerations related to myomectomy

In patients with prior myomectomy, the timing and route of delivery should be individualized based on the details of the previous uterine surgery, favoring cesarean for extensive or complicated myomectomy and allowing trial of labor for uncomplicated cases. Planned cesarean birth is a consideration in patients who have undergone prior transmyometrial myomectomy.^55^ Extensive myomectomy may be considered to be a myomectomy in which extensive or complete invasion of the myometrium is required for the removal of one or several intramural or submucosal fibroids of appreciable size.^25^ Cesarean birth before the onset of spontaneous labor is recommended for patients who have undergone extensive or complicated myomectomy, similar to those who have had a previous classical cesarean birth. The recommended delivery time for these patients is −36 + 0 to 37 + 0 weeks of gestation.^56^ Patients with less extensive prior surgery may be delivered as late as 38 + 6 weeks.^57^ For patients with prior, less extensive, uncomplicated myomectomy, management similar to patients who have had a prior cesarean is recommended. A trial of labor with continuous intrapartum fetal monitoring, early access to obstetric anesthesia, and the ability to perform an emergency cesarean should be carried out. Patients who have had a pedunculated fibroid do not typically require additional intrapartum monitoring if there is no other obstetric indication.^25^

The risk of uterine rupture after a myomectomy is not significantly greater than with vaginal birth after cesarean. A systematic review showed that the overall incidence of uterine rupture after myomectomy was 0.93% (95% CI, 0.45–1.92). The incidence was 0.47% (95% CI, 0.13–1.70) in patients undergoing trial of labor after myomectomy and 1.52% (95% CI, 0.65–3.51) in those with planned cesarean before the onset of labor; this difference was not statistically significant.^58^

A previous cesarean birth and transmyometrial myomectomy or any other causes of myometrial damage (e.g. prior hysteroscopic removal of a submucosal fibroid) may increase the risk of abnormal placentation, especially placenta accreta. Clinical research data on the association between preconceptional myomectomy and abnormal placentation are controversial. Although the risk of developing placenta accreta spectrum diseases seems to be low, it is recommended to perform an ultrasound examination to check for possible placenta accreta spectrum during the late second or early third trimester.^25^

### 7.3 Operative challenges during cesarean birth

Cesarean birth in patients with large, anterior, lower uterine segment fibroids is associated with a high risk of intrapartum or postpartum hemorrhage. The location of the hysterotomy incision and fetal extraction can be complicated; therefore, appropriate preparations should be made before the operation.

Antepartum ultrasound should be used to specify fibroid location, fetal presentation, and fetal position. The optimal location for the hysterotomy incision should be identified in advance. In some cases, intrapartum ultrasound may be utilized to assist in this process. During the procedure, a sterile probe cover is placed on the ultrasound transducer and sterile gel is applied to the uterine serosa. This allows for sonographic mapping of the incision site to determine the best location for the incision.^25^

To reduce the risk of postpartum hemorrhage, it is recommended that hemoglobin levels reach at least 9.5–10 mg/dL before the procedure. Blood products and a cell saver should be prepared prior to the operation. The standardized surgical protocol recommendations for patients at risk of complex cesarean may be beneficial in complicated cases.^59^ Additionally, the preoperative placement of bilateral iliac artery balloon catheters may be considered on a case‐by‐case basis.^44^

To create sufficient space for the procedure, a vertical skin incision may be necessary. A hysterotomy should be performed to prevent cutting through a fibroid and to select the most accessible incision line, ensuring it is at least 2 cm away from the edge of the fibroid. If feasible, a lower segment cesarean is preferred.^44^ A posterior classical hysterotomy often requires J‐shaped or U‐shaped incisions to avoid large fibroids located in the lower uterine segment. If the obstetrician struggles to accurately identify the anatomical landmarks for hysterotomy due to uterine rotation, the patient's uterus may be pulled outside of the abdomen.^60^

Cesarean myomectomy should generally be avoided. Patients undergoing this procedure experience a greater drop in hemoglobin (mean difference of 0.25–0.27 mg/dL), an approximately 40% increase in blood transfusions, and a longer hospital stay.^61^, ^62^ Pedunculated subserosal fibroids may be safely removed during cesarean without significantly increasing hemorrhage risk.

Observational data indicate that cesarean myomectomy can be performed without a high risk of life‐threatening complications, provided the obstetrician possesses the necessary expertise, appropriate patients are selected (such as those with symptomatic pedunculated fibroids), and blood products are readily available.^61^, ^62^ This decision should be made carefully, considering the biases that are inherent in observational studies.^25^

## 8 POSTPARTUM EVALUATION

Fibroids typically decrease in size after pregnancy. Research shows that 90% of patients who have fibroids identified in the first trimester will experience a reduction in total fibroid volume. However, about 10% of patients with fibroids may see an increase in total fibroid volume when re‐evaluated 3–6 months after birth.^20^ Evaluating fibroids is important to assess any significant increases in the size or number of fibroids, as this may affect future reproductive health or necessitate intervention.

Even after pregnancy, fibroids can sometimes cause pelvic pain, which can affect a patient's quality of life. Similar to during pregnancy, hormonal changes or reduced blood supply to the fibroid after birth may lead to degenerative changes. Additionally, necrosis can increase the fragility of the tumor, which may result in the rupture of the degenerated fibroid.^63^

When a myoma becomes infected, it can lead to a condition known as pyomyoma. Although this is a rare occurrence, pyomyoma should always be considered in cases of postpartum fever, particularly following intrauterine procedures like curettage or cesarean.^64^ Symptoms of pyomyoma often include fever, abdominal pain, and a palpable abdominal mass. If left untreated, it can develop into a life‐threatening condition due to complications such as peritonitis or sepsis.^64^

During routine postpartum assessments, it is important to evaluate fibroids and any changes related to them, especially if the patient does not experience any symptoms associated with fibroids in the postpartum period.

## 9 CONCLUSIONS

Pregnancies in patients with fibroids are generally uncomplicated. Complications tend to arise when there are multiple fibroids, when they are larger than 5 cm, or when they are located in the lower uterine segment. While these issues can be prognostic, the placement of the placenta in relation to the fibroids is unpredictable.

Management of patients with fibroids attempting to conceive should be individualized, considering factors such as patient age, reproductive history, severity of symptoms, and the number, size, and location of fibroids. Decisions regarding whether to perform a myomectomy or other interventions—such as embolization or high‐intensity focused ultrasound—or to proceed with pregnancy despite the presence of fibroids should be based on individualized clinical assessment.

There is a lack of consistent data on the timing of conception after myomectomy and limited evidence regarding the outcomes of cesarean myomectomy. As a result, research on these topics should be prioritized. Additionally, it is essential to conduct prospective studies on fibroid regression after childbirth.

## AUTHOR CONTRIBUTIONS

DR and BJ contributed to the conceptualization, planning and design. DR, GKB, NP, ID, TKB, and JBK contributed to the literature search, review, and synthesis. DR wrote the first manuscript draft. BJ and JBK reviewed, revised, and edited the first manuscript draft. BJ coordinated the reviews, revisions, and edits while DR implemented the reviews, revisions, and edits to produce a final manuscript. All authors read and agreed with the final manuscript.

## FUNDING INFORMATION

No funding was received.

## CONFLICT OF INTEREST STATEMENT

The authors have no conflicts to declare.

## Data Availability

Data sharing is not applicable to this article as no new data were created or analyzed in this study.
